# A transcriptome-wide analysis deciphers distinct roles of G1 cyclins in temporal organization of the yeast cell cycle

**DOI:** 10.1038/s41598-019-39850-7

**Published:** 2019-03-04

**Authors:** Lotte Teufel, Katja Tummler, Max Flöttmann, Andreas Herrmann, Naama Barkai, Edda Klipp

**Affiliations:** 1 Theoretical Biophysics, Humboldt-Universität zu Berlin, Berlin, Germany; 2 Molecular Biophysics, Humboldt-Universität zu Berlin, Berlin, Germany; 3 Weizmann Institute of Science, Rehovot, Israel

## Abstract

Oscillating gene expression is crucial for correct timing and progression through cell cycle. In *Saccharomyces cerevisiae*, G1 cyclins Cln1–3 are essential drivers of the cell cycle and have an important role for temporal fine-tuning. We measured time-resolved transcriptome-wide gene expression for wild type and cyclin single and double knockouts over cell cycle with and without osmotic stress. Clustering of expression profiles, peak time detection of oscillating genes, integration with transcription factor network dynamics, and assignment to cell cycle phases allowed us to quantify the effect of genetic or stress perturbations on the duration of cell cycle phases. Cln1 and Cln2 showed functional differences, especially affecting later phases. Deletion of Cln3 led to a delay of START followed by normal progression through later phases. Our data and network analysis suggest mutual effects of cyclins with the transcriptional regulators SBF and MBF.

## Introduction

Eukaryotic cell cycle is a highly ordered process, which can be divided into four distinct phases during which a specific set of events take place: Cell growth (G1 and G2 phase), duplication of DNA (S phase), the segregation of DNA and the division of the nucleus (M phase), finally leading to cytokinesis. To enable and control the progression through the cell cycle, a subset of genes is transcribed in an oscillating pattern. Amongst those, cyclins are key regulatory proteins, which trigger all fundamental events of the cell cycle^1,2^. Cyclins activate cyclin dependent kinases (CDKs), leading to phosphorylation of specific target proteins, which among other things, initialize the expression of the next wave of oscillating genes (for simplification we will refer to the cyclin-CDK complexes just by the name of their cyclins). The cyclins are functionally conserved across many species including mammals^3^, which makes understanding their functions even more relevant.

During G1 phase, the initial part of the cell cycle, three cyclins in *Saccharomyces cerevisiae* are necessary to successfully start the first regulatory events of the cell cycle: Cln1, Cln2 and Cln3. These G1 cyclins have been extensively studied and were found to fulfill specific functions. Knockout studies have shown that they are able to partly compensate for each other^4–7^. However, loss of all three cyclins at once is lethal for the yeast cells^7^, highlighting their importance for controlling cell cycle progression.

**Figure 1:**
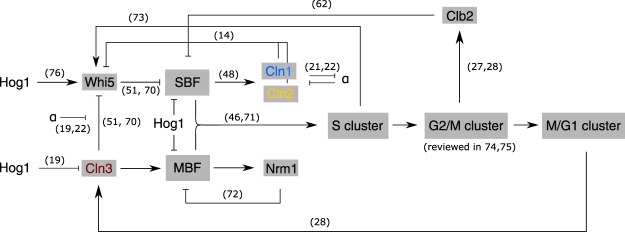
Wiring diagram of the cell cycle, based on references^14,18,20–22,27,28,46,48,51,62,70–76^ and results of this work. Main mechanisms of oscillating gene activation and inhibition are represented. G1 cyclins Cln1, Cln2 and Cln3 are shown in blue, yellow and red, respectively. G1 regulon (MBF/SBF) activation is shown in detail, activation of the following gene clusters (S, G2/M and M/G1 cluster) are only shown as schematic overview. Activation is represented as arrows and inhibition as a bar-headed arrow. In addition, the effects of α-factor and osmotic stress are depicted. Related publications for each regulatory edge are shown as numbers next to the arrows.

Cln3 is the first cyclin expressed during G1 phase, which, by inactivating the transcriptional repressor Whi5, is responsible for cell size control and the initial expression of the G1/S regulon. Two hypotheses have been proposed for the mechanism of the Cln3-Whi5 interaction: Both, either the retention of Cln3 in the endoplasmic reticulum with release in late G1 phase^8,9^ or the size-dependent dilution of Whi5 up to a critical threshold^10^^,^ can explain the Cln3-mediated release of Whi5 repression at the right time to initialize the transcription of genes required for the G1/S transition. In both cases, once Whi5 is sufficiently phosphorylated by Cln3, it is excluded from the nucleus and two transcriptional complexes, MBF (Mlu1 Cell Cycle Box [MCB] Binding Factor), consisting of Swi6 and Mbp1, and SBF (Swi4/6 cell cycle box [SCB] Binding Factor), a heterodimer of Swi6 and Swi4^11,12^, can trigger the expression of genes in the G1/S regulon. However, the detailed wiring of this phase of the cell cycle network is still debated (for references see Fig. 1).

The cyclins Cln1 and Cln2 are expressed in the G1/S regulon. They share the same wiring in the cell cycle network and are structurally highly similar^13^, which is why they are usually considered to carry out the same functions. In a positive feedback loop, both cyclins contribute to further phosphorylation of Whi5, thereby increasing the activation of MBF and SBF controlled genes and also their own expression^14^. Besides this self-enhancement, Cln1 and Cln2 phosphorylate further targets, such as the S phase inhibitor Sic1^15,16^, leading to its degradation and a subsequent entry into S phase in the continuing cell cycle.

This transition from G1 to S phase is called START and marks a point of no return. Hence, if a cell overcomes this checkpoint and commits to entering S phase, it has to progress through the entire cell cycle. Accordingly, this checkpoint has to be tightly controlled to ensure that the cell is well prepared for a save passage to cell division. Many regulatory processes and pathways are therefore active before the checkpoint to control for both internal factors, such as cell size, genomic integrity or availability of storage compounds, and external conditions, such as available nutrients or environmental stresses. Many of these stresses induce a cell cycle arrest in G1 phase, which can be released to pass the checkpoint only after the stress has been counteracted by the cell. Well known examples are the response to an increase in external osmolarity or, in haploid cells of mating type *MAT***a**, the arrest due to the mating pheromone α-factor^17^. Both processes are also known to interact with the G1 cyclins: Osmotic stress, via the Hog1 signaling pathway, inhibits *CLN1* and *CLN2* expression^18^ and Cln3 activity^19^, whereas the pheromone pathway component Far1 is known to be a target of Cln1 and Cln2^20–22^. The G1 cyclins are, therefore, key not only to normal cell cycle progression but also to the control of the cell cycle in stress scenarios.

In this work, we aim to dissect the specific functions of the three G1 cyclins in fine-tuning of cell cycle timing. In particular, we are interested in understanding the contribution of each cyclin to organizing the passage through G1 phase and to identify effects on later cell cycle phases. A major question is thereby whether Cln1 and Cln2 are really fully redundant or whether we can identify specific influences on the cell cycle timing for each of them. To do so, we characterized the roles of the G1 cyclins in organizing global oscillatory gene expression. We analyzed the transcriptome of wild type as well as single and double knockouts of Cln1, Cln2 and Cln3 to identify timing effects, such as overall delays or temporal shifts, in the expression patterns. For enhancing functional differences between Cln1 and Cln2, we additionally perturbed the cells with osmotic stress. Based on the results, we propose mechanistic links that could cause the observed timing effects depended on altered transcription factor activities.

We found that the deletion of the earliest cyclin Cln3 leads to a systematic shift of expression times, with an elongated G1 phase, especially under osmotic stress condition and in combination with the knockout of Cln1, followed by a wild type like timing of the subsequent phases of the cell cycle. Loss of Cln2, on the other hand, results in an elongated G2 phase following a relatively conserved initial cell cycle. These timing effects correlate to changes in the activation of the G1 regulon triggered by MBF or SBF. Losing Cln1 has a less dramatic effect than a Cln2 knockout, which only becomes evident when additional stresses are present: *cln1*Δ cells require longer times to exit from G1 phase after an osmotic shock and Cln1 alone (without Cln2 and Cln3) is not able to induce oscillating gene expression. Taken together our results show that the correct activation of the G1 regulon and thereby induction of oscillating gene expression is strongly dependent on the G1 cyclins, each having specific roles in the adjustment of cell cycle timing.

## Results

### Knockout of G1 cyclins alters cell size and transcriptional timing

To understand the mechanistic contribution of the major G1 cyclins Cln1–3 to the orchestration of the cell cycle, we analyzed the effect of their loss in single and double mutants compared to the wild type strain, BY4741, a haploid strain of mating type *MAT***a**. Those mutants are, as opposed to the triple mutant^7^, viable and allow for characterization of specific effects of the loss of each cyclin.

**Figure 2:**
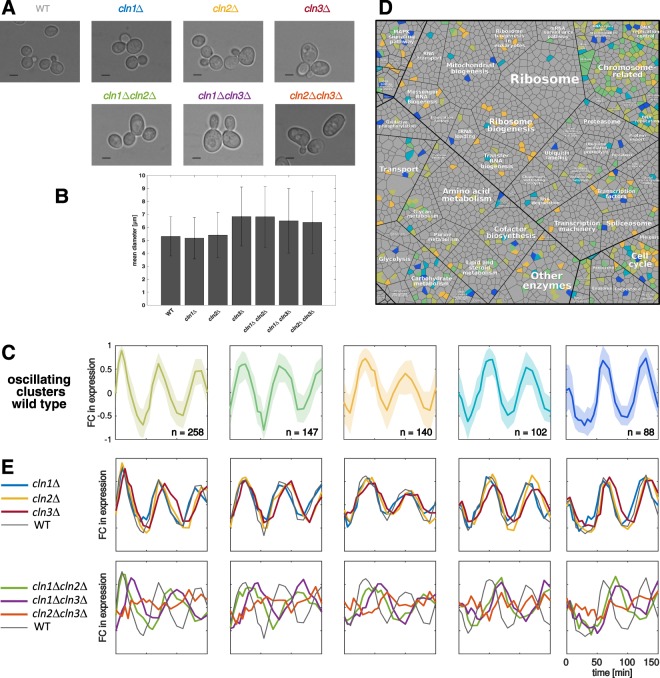
Characterization of cyclin deletion mutants. (**A**) Brightfield microscopic images of the used strains. Scale bar represents 4μm. (**B**) Cell sizes (diameter in μm) and standard deviation quantified by CASY® Cell Counter of wild type (WT) and knockouts are shown (in total approximately 200000 cells were measured). Welch test was performed to test significance of size differences for the deletion mutants compared to the wild type. We found a significant difference in size for all strains w.r.t the wild type (*P* < 0.05). They showed size differences about 0.14 µm for *cln1Δ*, −0.11 µm for *cln2Δ*, −1.53 µm for *cln3Δ*, −1.51 µm for *cln1Δcln2Δ*, −1.2 µm for *cln1Δcln3Δ* and −1.08 µm for *cln2Δcln3Δ*. The effect size was low for *cln1Δ* and *cln2Δ* with 0.088 and 0.066, respectively and higher for *cln3Δ* with 0.81 and the double mutants *cln1Δcln2Δ*, *cln1Δcln3Δ* and *cln2Δcln3Δ* with 0.76, 0.57, 0.53, repectively. **(C)** Wild type expression of oscillating gene clusters, obtained by k-means clustering, sorted according to their peak times (fold change to mean of each gene, line represents mean of the cluster genes’ expression, shaded area 25% and 75% quantiles). **(D)** Functional classification of the oscillating genes based on a proteomap^29^. Each tile represents a gene, grouped according to its product’s function. Genes contained in the oscillating clusters in C are highlighted in the corresponding colors. **(E)** Mean expression of the oscillating gene clusters in the mutant strains in single mutants (upper panel) and double mutants (lower panel). For comparison, the wild type (WT) behavior is plotted in gray.

As was shown before^23,24^, Cln genes act as regulators of cell size and morphology (Fig. 2A). The loss of two out of three G1 cyclins causes the cells to increase in size compared to the wild type before division (Fig. 2B). While the loss of Cln1 or Cln2 leads to a wild type like size, the loss of the initial cyclin Cln3 as single deletion already causes an increase in cell size comparable to the double deletions.

Changes in cell size can hint towards a deregulation of cell cycle timing, with altered cell cycle phase durations resulting in longer or shorter growth periods. We, therefore, set out to characterize the specific timing of cell cycle events. Progression through the cell cycle can be well monitored by the periodic expression of genes in specific cell cycle phases^25–28^. Accordingly, we performed time-resolved RNA sequencing (RNAseq) on the mutant strains following the release from synchronization with α-factor. To identify genes that showed an oscillatory behavior, we applied k-means clustering on the wild type gene expression trajectories (Fig. 2C and Supplementary Fig. S.1). Oscillating genes were grouped in five clusters with different peak times throughout the cell cycle. The wild type, thereby, showed two complete cell cycle periods within 150 minutes. A functional classification of the genes in the oscillating clusters showed that they take part in a wide variety of cellular processes (based on a generic proteomap^29^, Fig. 2D). GO term analysis^30,31^ further revealed ordered timing of cell cycle functionalities for the wild type: Genes for DNA replication and chromosome organization (cluster 1) are followed by genes involved in chromosome segregation, chromatin assembly and microtubuli organization (cluster 2), subsequently in nuclear division and mitotic exit (cluster 3 + 4) and finally by genes required for the next cell cycle such as G1/S transition genes (cluster 5, for detailed results see Supplementary File 1).

The well-characterized oscillatory gene subset in the wild type could be used to examine the transcriptional changes occurring in the mutant strains (Fig. 2E). The oscillating behavior of the gene clusters was conserved in the mutants except for the *cln2*Δ*cln3*Δ strain that did not show any oscillatory gene expression (lower panel, orange line). All other strains’ gene expression still oscillated, but showed timing effects of different magnitudes. While in the single knockouts we found no or only moderate changes in period and timing of the oscillations (upper panel), we observed strong delays and relative shifts in the double mutants (lower panel). Qualitatively, mutants lacking Cln3 showed a delayed expression in all five clusters, arguing for a delayed onset of the cell cycle, whereas the loss of Cln2, especially in combination with Cln1, only delays the expression of later clusters (2–5), hinting towards a deregulation of individual cell cycle phase lengths.

### Cln mutants maintain transcriptional oscillations but not their timing

The clustering analysis revealed a qualitative overview of the implications of G1 cyclin loss. Quantifying these effects on the level of individual genes allowed us to systematically characterize specific timing effects of each mutant, such as extensions of time intervals of each phase within the cell cycle.

**Figure 3:**
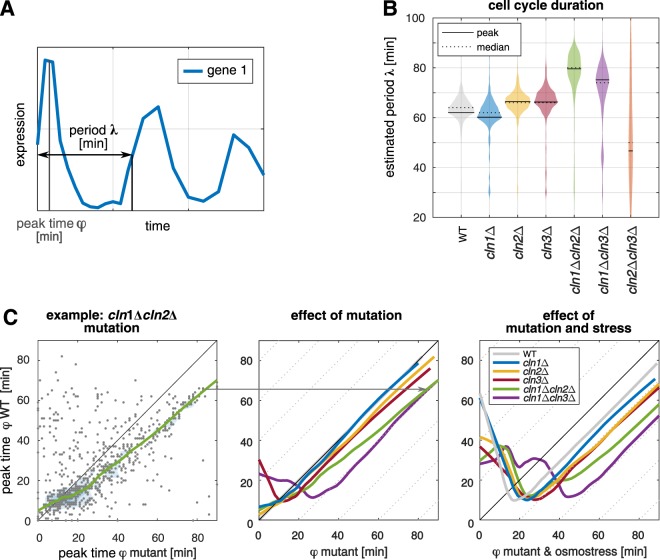
Quantitative peak time analysis. (**A**) Schematic representation of the oscillation properties of a gene, as estimated by MoPS algorithm^25^. (**B**) Estimated period λ, corresponding to the cell cycle duration, of wild type (WT) and mutants (solid line represents most frequent period and dotted line represents median of period) as estimated using the MoPS algorithm^25^. (**C**) Shift in estimated peak times ϕ. Left: as example *cln1Δcln2Δ* is shown. All genes contained in the oscillating clusters for mutants w.r.t. the wild type expression (y-axis) are presented. Each gray dot represents one gene, colored lines are lowess smoothed curves. While genes occurring at the bisecting line have the same peak time in mutant and wild type, genes below the bisecting line have higher peak times in the mutant. Middle: Effect of mutations on peak time ϕ w.r.t. wild type, lowess smoothed curves for all mutants except of cln2Δcln3Δ are shown. Right: Combined effect of mutation and osmotic stress compared to unstressed wild type.

Based on the expression data, we estimated the peak time **φ** of all measured genes with the help of the MoPS algorithm^25^ (Fig. 3A). The peak time estimates have a higher time resolution than our sampling intervals, such that we could also estimate peak times that lie between two probed time points. The algorithm furthermore provides a scoring of periodicity (see Material and Methods and Supplementary Fig. S.2) as well as an estimate for the period λ of the oscillations (Fig. 3A), which corresponds to the cell cycle length. The median cell cycle duration of the so identified subset of oscillating genes (Supplementary File 2) was 64 minutes in the wild type (Fig. 3B). The single deletion mutants only showed a slightly changed cell cycle duration The double deletions *cln1*Δ*cln2*Δ and *cln1*Δ*cln3*Δ showed a clear extension by 16 min and 10 min, respectively, of the cell cycle compared to the wild type (Fig. 3B) in accordance with their increased cell size. Due to the loss of oscillating gene expression, cell cycle duration could not be estimated with this method in the *cln2*Δ*cln3*Δ strain.

The estimated peak times of the individual oscillating genes were used to systematically analyze quantitative differences in the expression timing. For each mutant, peak times were compared to the wild type estimates in a two-dimensional scatterplot (as an example *cln1*Δ*cln2*Δ is shown in Fig. 3C, left). We applied lowess smoothing to better visualize the general behavior of the entire group of oscillating genes. As genes occurring close to the bisecting line have a conserved peak time in mutant and wild type, we can use the slope of the smoothed curve to identify delays in specific time intervals (characterized by slopes smaller than one). Shifted curves with a conserved slope around one indicate an overall delay with a conserved peak timing, for example caused by a delayed onset of the cell cycle.

With all peak times gathering around the bisecting line, no effect of the loss of Cln1 alone on the overall cell cycle timing was evident (Fig. 3C, middle, blue line). In contrast, cells lacking Cln2 (as single deletion (yellow) or in combination with Cln1 deletion (green)) showed a conserved timing of the early peaks, but a decreased slope for later times – arguing for a slower progression through later cell cycle phases such as S and G2/M phase. In the *cln1*Δ*cln3*Δ double mutant (purple) and, slightly less prominent, in the *cln3*Δ single deletion (red), we observed that the overall sequence of the peak times was retained (slope close to one) with a delay of around 20 and 5 minutes, respectively. However, a subset of genes shifts their peak time backwards to the first third of the cell cycle, visible as a negative slope of the lowess curve. A thus elongated G1 phase, in which many genes show their peak expression, is a behavior often observed in cells that react to a stress stimulus.

Following this thought, we repeated the peak time analysis with cells that had been exposed to osmotic stress following their release from synchronization. Osmotic stress is known to arrest cells in the G1 phase of the cell cycle until the stress is counteracted^18,19,32–34^. Arrest in later phases is neglected here, since cells were exposed to osmostress in early G1. Since also our target cyclins act primarily in the G1 phase, we hypothesized that an additional perturbation in this phase could reveal further aspects of their action. In the stress experiments, we observed a delay of oscillating gene expression for wild type and all mutants (Fig. 3C, right). Also in the *cln1*Δ strain, which did not show changes in expression timing under unstressed conditions, we could now observe a stronger delay than in the wild type (11.5 mins delay caused by osmotic stress in the wild type, 16.9 mins in *cln1*Δ), highlighting the role of Cln1 in reentering the cell cycle after osmostress.

In summary, the peak time analysis revealed specific characteristics of the mutant gene expression: The loss of Cln3 causes an overall delay in oscillating gene expression, while the loss of Cln2 results in a relative shift of peak events towards later cell cycle times. Furthermore, by means of stress experiments, we could demonstrate that Cln1 and Cln2 are functionally non-redundant, whereby loss of Cln1 causes deregulation of transcriptional timing under osmostress.

### The network of periodically acting transcription factors reveals phase specific timing effects

The observed timing differences already contributed to the understanding of Cln functionality, but so far lacked mechanistic explanation. Our RNAseq data provided a characterization of the timing of gene expression, which is usually the result of the action of transcription factors. We, therefore, analyzed the systemic differences in transcription factor action in the mutants to wild type in order to identify candidate factors responsible for the timing effects.

**Figure 4:**
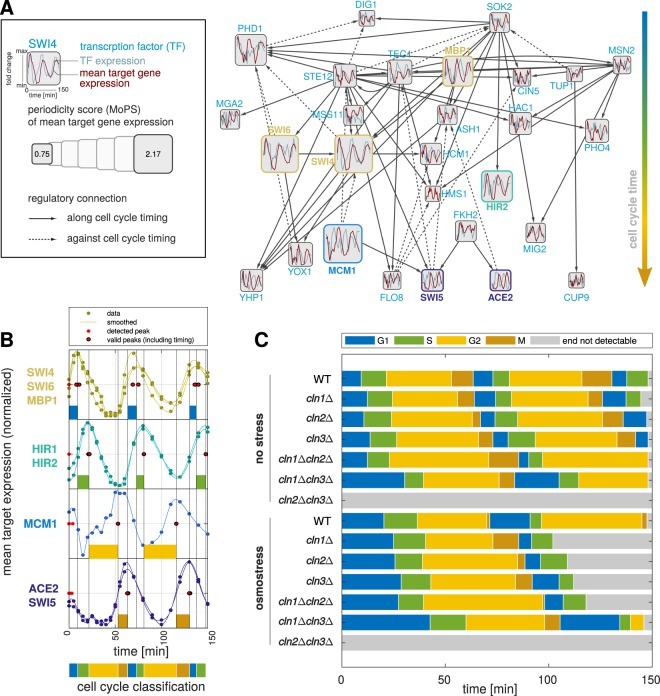
Cell cycle phase classification by active transcription factor (TF) network. (**A**) Summary of the oscillating transcription factor network in the wild type (WT). Vertical arrangement follows the target gene peak times (TFs with early peaking targets at the top). Regulatory edges that adhere to the cell cycle timing are shown as solid lines (start-point TF’s targets peak earlier than end-point targets) and as dashed lines otherwise. (**B**) Wild type target expression of “classification set” transcription factors used to define cell cycle phase durations, trajectories depict mean expression level of the targets (log fold change to mean). Phase transitions were defined according to the rules in Supplementary Table S.2. (**C**) The accordingly classified phases of all strains and conditions. See also Supplementary Fig. S.4. For further analysis, we considered G2 and M phases together.

We utilized a generic transcription factor network (YEASTRACT^35^, Supplementary File 3) and reduced it to the oscillating subnetwork of our interest. To do so, we used the MoPS algorithm^25^ to calculate the periodicity scores of the mean trajectory of all differentially expressed targets of each transcription factor in the wild type dataset. We discarded all transcription factors with a target periodicity score smaller than 0.75 or with less than three target genes. In the resulting, fully oscillatory network (Fig. 4A, Supplementary Table 1) many transcription factors are present that are known to be involved in the cell cycle. Consistent with the expected information flow in the network, most of the regulatory edges connecting the oscillating transcription factors are directed from early to later peaking genes (top to bottom in Fig. 4A), only some are in the reverse direction (*e.g*. from Ace1, Swi4, Ste12, Tec1). These backward regulations could either be explained by inhibitory function of the transcription factor or by regulation of early genes in the following cell cycle.

Based on this wild type regulatory network, we could identify transcription factors that showed a changed temporal expression in the mutants. To reduce complexity, we focused on the transcription factors with the highest periodicity score. Thereby, we selected factors representative for specific cell cycle times, which often corresponded to transcription factors known to be active at the transition between cell cycle phases. Our final “classification set” of transcription factors (Supplementary Table 2) consists of four groups, whose peak expression occurs at the transitions between G1/S, S/G2, G2/M and M/G1 phase (wild type example in Fig. 4B, mutants in Supplementary Fig. S.4). Based on a variability analysis carried out with 5 replicates of the wild type gene expression, we could identify the lengths of G1, S and G2/M phase with an accuracy of 5 minutes (Supplementary Fig. S.12), which we further on used as minimum detectable change in phase lengths. From the classification set, we estimated the cell cycle phase durations in all stressed and unstressed mutants (Fig. 4C). It is important to notice that we detected the peak expression times here with a simple peak detection based on the smoothed target trajectories as opposed to using the MoPS peak time estimates. The reason for this is that especially for the stress experiments, but also due to the influence of the α-factor synchronization, the first measured cell cycle in our experiment can have different phase lengths than the second one. The MoPS algorithm^25^, however, uses one fixed period for the entire time course. While this was not a problem in our previous analyses, which aimed at identifying oscillatory genes, we are now interested in the specific shifts in the phase timing. In our analysis, we focused on the first cell cycle since α-factor and osmotic stress mainly interact with the first cell cycle and synchrony decreases during the second cell cycle.

Due to the lack of oscillations, no cell cycle phases could be assigned for *cln2*Δ*cln3*Δ, while we could identify all phases as well as specific timing effects for the other strains. While the single mutants showed only slight alterations in the length of the first G1 phase, strong effects were detected in the double mutants. *cln1*Δ*cln3*Δ showed the longest G1 phase, which is without stress longer than the osmotically stressed wild type G1 phase. In contrast, not only G1 but also G2/M phase were extended in the *cln1*Δ*cln2*Δ strain. It is known that cells grow especially in G1 phase and that a G1 arrest leads to bigger cells^14,36,37^. This is supported by our results showing that cells with extended G1 phase, like the double deletion *cln1*Δ*cln3*Δ, were significantly bigger than the wild type (Fig. 2B). Additionally, our data showed that an increase in size is not only dependent on the duration of G1 but also of G2. That is demonstrated, for example, by size differences of *cln1*Δ and *cln1*Δ*cln2*Δ both having similar G1 phase duration (estimated period for both mutants 12.3 min), while the double deletion has a much longer G2/M phase than the single mutant (estimated periods *cln1*Δ: 31.5 min, *cln1*Δ*cln2*Δ: 48 min), corresponding to a bigger cell size of *cln1*Δ*cln2*Δ.

The addition of a high concentration of osmolytes arrests yeast cells in G1 phase, until cells finish their stress response and are once again ready to commit to a new cell cycle^18,19^. This additional elongation of the G1 phase was of comparable magnitude in the single mutants and elongated in the *cln1*Δ*cln3*Δ strain. In the stress experiment, also the *cln1*Δ strain showed an elongated cell cycle compared to the wild type.

The individual phase lengths defined by the oscillating transcription factor network allowed us to dissect the observed timing effects: Cells lacking Cln3 spend longer time in G1 phase, in which the expression triggered by the relevant transcription factors (Swi4, Swi6, Mbp1) rises less rapidly than in the remaining mutants. In cells lacking Cln2, the shifted peak times occurred in an extended G2/M phase, which was marked by a delayed expression of the targets of the later transcription factors Mcm1 and Ace2/Swi5.

### Loss of CLN3 synchronizes SBF and MBF target expression

For the Cln3 deletion mutants, we observed altered expression in the first group of transcription factors (Swi4, Swi6, Mbp1) from our classification set. Those proteins are known to form the functional complexes MBF and SBF, which critically regulate the expression of the G1/S regulon and, thereby, facilitate the transition between G1 and S phases^35,38–43^. Functionally, MBF targets are mostly involved in organizing DNA replication and adjunct processes while SBF targets control cell morphology^44–46^. Cln1–3 are involved in the regulation of MBF and SBF and, in the case of Cln1 and Cln2, are also themselves part of the G1/S regulon^47,48^. MBF and SBF are regulated by slightly different mechanisms that share part of their actors. The explicit roles of the cyclins in this regulation, especially for MBF, are, however, still unknown.

**Figure 5:**
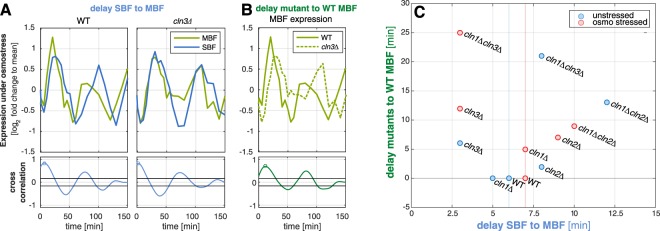
Detailed view on MBF and SBF target gene expression. (**A**) Delay between MBF (green line) and SBF (blue line) target expression, shown exemplary for wild type (WT) and *cln3Δ* (target list of MBF/SBF obtained from YEASTRACT^35^. Upper part shows the mean expression of all target genes (normalized as log2 fold change to the temporal mean of each gene), lower part indicates the cross correlation of the two trajectories, the maximum is marked with a circle. (**B**) Delay of MBF target gene expression in a mutant (as example *cln3Δ* is shown, dotted line) to the wild type expression (solid line). Upper and lower panels as in A. (**C**) Summary of the measures exemplified in A and B for all mutants under both unstressed (blue dots) and osmostressed (red dots) conditions. The delay between SBF and MBF expression is shown on the x-axis, the delay of the mutant to the wild type MBF expression on the y-axis.

With the time resolved transcriptomics data, we could analyze the expression pattern of the two transcriptional complexes’ target genes in detail. We used the list of target genes provided by YEASTRACT^35^. We calculated two quantitative measures based on auto-correlations: (i) the delay between the expression of SBF and MBF target genes in each mutant (Fig. 5A) and (ii) the delay of MBF target gene expression in each mutant compared to the wild type expression (Fig. 5B).

In α-factor synchronized cells, MBF targets are expressed some minutes before SBF targets^40^. We observe the same behavior in the wild type, in which the SBF peak occurs around 6 minutes after the MBF peak. Also our mutant strains showed the same temporal order, but the delays between the two target cluster expressions were affected by the mutations (Fig. 5C). The interval between the MBF and the SBF peak expression is longer in mutants lacking Cln2 (estimated delay 8 min and 12 min for *cln2*Δ and *cln1*Δ*cln2*Δ, respectively). Contrarily, in cells lacking Cln3, MBF expression itself was delayed strongly compared to the wild type, whereas the interval between MBF and SBF expression was shortened (estimated ~3 min in the *cln3*Δ). Applying osmotic stress these observed behaviors becomes even more pronounced.

Because the assignment of MBF and SBF target genes is not unique and partially contradictory, we repeated the analysis based on three further published target lists (Supplementary Fig. S.5, target lists in Supplementary File 4,^35,39,40,49^). The obtained results were qualitatively reproducible for all analyzed target lists, even though they showed differences in the absolute values for the calculated delays. Especially, the *cln1*Δ*cln3*Δ strain showed a stronger synchronization of SBF and MBF target expression in the target lists not stemming from YEASTRACT^35^. Even though the shifts were reproducible for different target sets, these shifts should be experimentally verified especially in single deletion experiments where the peak shifts are smaller than our sampling time scale.

Focusing on the estimated cell cycle phase durations, our data suggests that we could link the extended G1 phases of the Cln3 mutant strains to the delayed expression of the MBF gene cluster. Accordingly, Cln3 has to have a - direct or indirect - role in the activation of MBF, which has been proposed before^50–52^. The observed elongation of the later G2/M phase, on the other hand, occurred in the strains that show a relative delay of the SBF cluster to the MBF cluster.

### *cln2*Δ*cln3*Δ cells show diminished silencing of α-factor dependent gene expression

**Figure 6:**
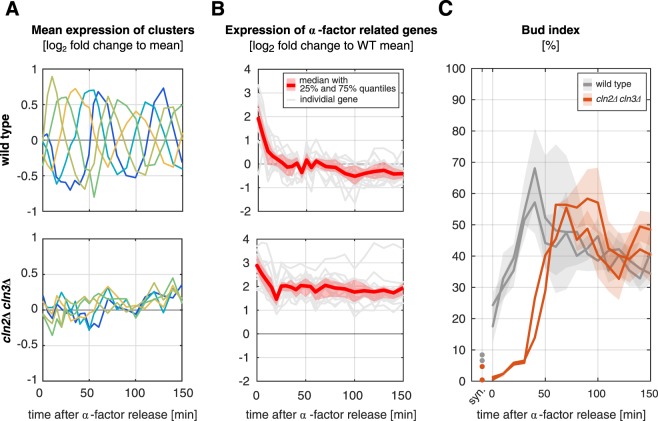
*cln2Δcln3Δ* cells fail to inhibit α-factor induced gene expression. (**A**) Expression of genes in the oscillating clusters (wild type (top panel) and *cln2Δcln3Δ* (bottom panel), same as in Fig. 2C and E). (**B**) Group of α-factor induced genes showing higher expression in *cln2Δcln3Δ* than in wild type, red line represents mean expression with 25% and 75% quantiles, gray lines represent individual genes (see also Supplementary Table S.3). (**C**) Timing of bud appearance following release from α-factor. Two biological replicates with more than 200 cells per time point are shown for wild type (gray) and *cln2Δcln3Δ* (orange). Shaded areas represent uncertainties due to unsure splitting of cells (see materials and methods). Time point “syn” was measured after 3 h of α-factor synchronization.

Our results outline a predominant influence of Cln3 and Cln2 on the timing of the cell cycle phases. Consistently, but complicating for further analysis, the *cln2*Δ*cln3*Δ strain did not show oscillations in gene expression (Fig. 6A), indicative of a disruption of cell cycle timing. Nevertheless, the mutant is able to grow and divide in culture (Fig. 2A), and to orchestrate an appropriate transcriptional response to osmotic stress (Supplementary Fig. S.6).

In the cluster analysis, we also found a cluster of genes whose expression is highest at the beginning of the experiment and decreases continuously afterwards. The cluster behavior was robust in all mutants, but not in the *cln2*Δ*cln3*Δ strain (Supplementary Fig. S.1, cluster 4), where its expression stayed at a much higher level. Based on functional enrichment of genes for conjugation, sexual reproduction, cell aggregation and cellular response to pheromones in this cluster (Supplementary File 1), we could characterize the behavior as a decaying response to α-factor arrest. Zooming in further, we identified a group of 16 genes related to α-factor signaling and its cellular response (Supplementary Table 3), which remained at levels 2.5–7.7-fold higher in the *cln2*Δ*cln3*Δ strain than in the wild type (Fig. 6C).

We therefore hypothesized that cells lacking both Cln2 and Cln3 are more susceptible to α-factor arrest and less efficient in adapting once the α-factor is removed from the medium. The cause for this is, however, not an asynchronous exit from the cell cycle arrest, which we assessed by DNA staining and FACS analysis as well as by the timing of budding events. The FACS measurements showed a delayed duplication of the DNA in the *cln2*Δ*cln3*Δ strain (~60 min compared to 20 min in the wild type, Supplementary Fig. S.7). The bud index confirmed this result, showing a synchronous but delayed occurrence of budding events (Fig. 6C, highest bud index at 60 min, compared to 40 min in the wild type). In conclusion, the de-synchronization which is evident by the lack of oscillations in the gene expression must stem from later phases of the cell cycle.

We, therefore, concluded that the double mutant lacking both Cln2 and Cln3 is less efficient at silencing α-factor signaling in a coordinated fashion once the pheromone is removed from the medium. The budding pattern as well as the FACS measurements indicate a delayed exit from G1 phase, whereby the budding events still occur within a narrow time frame (~50–60 min after removal of α-factor). Accordingly, as we do not detect any oscillations in the mRNA expression, the culture either loses synchrony in later phases of the cell cycle or starts to oscillate only after our measured time period of 150 minutes.

## Discussion

The G1 cyclins Cln1–3 are essential players in the initialization of the cell cycle (see schematic representation in Fig. 1). Based on transcriptome-wide, time-resolved gene expression data in different Cln knockout strains, we showed functional differences between them and explored their contribution to the fine-tuning of the cell cycle. Specifically, we showed that Cln1 and Cln2 have similar but clearly non-redundant functionalities, with Cln2 exerting stronger control over the cell cycle timing and influencing it beyond the initial G1 phase. Cln3, on the other hand, is known to have a distinct mechanism of action upstream of Cln1 and Cln2 activation. Consistently, we here characterized a strong and unique delay pattern in the *cln3*Δ mutant and proposed a MBF related mechanism for the observation. Consistent with the identified major roles of Cln2 and Cln3 in the start-up of the cell cycle, we show that a mutant lacking both genes fails to induce oscillating gene expression, related to an impeded down-regulation of α-factor signaling.

First, we showed distinct functions of Cln1 and Cln2 in the fine-tuning of the cell cycle. The two cyclins Cln1 and Cln2 are usually thought to have redundant function^6,21,53,54^, and in functional models of the cell cycle they are often lumped together in one species *e.g*.^55–58^. Experimentally, Cln1 and Cln2 show slight differences in expression timing^40^, degradation pattern^59^ and nuclear accumulation^13,60^, questioning a full redundancy of the two cyclins.

We found that loss of Cln2 had a more dramatic effect on the cell cycle timing than loss of Cln1. Cells lacking Cln2 showed a stronger shift in gene expression peak times (Fig. 3), a more prominent delay of SBF to MBF target gene expression (Fig. 5) compared to the *cln1*Δ mutant as well as a defective oscillatory capability in combination with the knockout of Cln3.

Both cyclins are part of a self-regulating positive feedback loop (Fig. 1). The identified functional differences finally allowed for the hypotheses that (i) repression of the transcriptional repressor Whi5 by Cln2 is stronger than by Cln1 and that (ii) the pheromone response is predominantly interacting with Cln2 (see below).

A surprising finding from our data was the elongation of the G2/M phase in cells lacking Cln2 (Figs 3, 4). In these mutants, we also observed a relative delay of SBF to MBF target gene expression. When the positive feedback loop via Cln2 is lost or reduced in its activity, the repression of SBF by Whi5 can only be relieved by the action of Cln3 (compare Fig. 1), which seems to be far slower, causing a delayed expression of SBF targets genes. A potential regulation of Swi4, which is part of the SBF complex, by MBF^61^ seems to only partly compensate for the action of the feedback. The delay in SBF expression could affect the length of later phases of the cell cycle, as observed here for the G2/M phase, via indirect links. For example, the transcription factor *NDD1* is under the control of SBF^49^. Ndd1 activates the transcription of a gene cluster including *CLB2* at the G2/M transition, which exerts a repression feedback on SBF^12,62,63^.

Furthermore, we found that the loss of Cln3 delays the onset of cell cycle and MBF activation. Amongst the single mutants, *cln3*Δ showed the strongest phenotype, including increased cell size and cell cycle duration, deregulation of oscillating gene peak times and number of differentially expressed genes (Supplementary Fig. S.8). The slope-conserving shift of all peak times in that mutant (Fig. 3) indicated an overall delay of the onset of the cell cycle, consistent with an elongated G1 phase (Fig. 4). Furthermore, we characterized a delay of MBF target gene expression in mutants lacking Cln3 (Fig. 5) that correlated with the delayed onset of the cell cycle. Taken together, these findings hint to a functional connection between Cln3 and MBF activation. From previous literature, the detailed mechanism of MBF activation is not yet known. It has been proposed that MBF de-repression at the start of the cell cycle is dependent on Cln/CDK but independent of Whi5^51^ (unlike SBF expression, which is dependent on both). Furthermore, Wittenberg and Reed hypothesized that MBF activation may involve direct phosphorylation of Swi6 or phosphorylation of another MBF associated protein^52^. Our results show that MBF expression is indeed dependent on the action of the G1 cyclins, but is not abrogated by the loss of any single one. However, the expression timing is influenced by the G1 cyclins, with the strongest effect caused by the loss of Cln3 (5–10 mins delay compared to the wild type, depending on the definition of targets, Supplementary Fig. S.5). In cells lacking Cln3, also the expression peaks of SBF and MBF are less delayed relative to each other. This more similar regulation of the two clusters in absence of Cln3 could be explained by a backup activation of MBF carried out by Cln1 and/or Cln2. Even though we found the same behavior for different target lists of MBF and SBF, it should be noted that these shifts are in a minute time scale (i.e. shorter than the periods between our samples) and, therefore, these results should be experimentally verified e.g. by measuring activity of the two transcription factors.

We additionally showed that the release from α-factor requires Cln2 and Cln3. The interference of α-factor synchronization with our knockout experiments first appeared to be a substantial drawback in our experimental setup. However, it allowed us to dissect an unexpected aspect of G1 cyclin action: their specific interaction with the pheromone-induced cell cycle arrest.

We showed that while cells lacking Cln2 as well as Cln3 are still able to exit from the arrest in a coordinated, but strongly delayed manner, the population behavior is desynchronized on the level of mRNA expression with no apparently oscillating genes. The de-synchronization must hence occur in later stages of the cell cycle. Alternatively, the lingering effect of α-factor could be so strong that oscillations become only visible much later than the 150 min measured in our experiments. This effect again highlights the higher importance of Cln2, compared to Cln1, in the timing of cell cycle progression, as this effect did not occur in the *cln1*Δ*cln3*Δ strain.

We can include *cln2*Δ*cln3*Δ to a previously described partially viable group of mutants^58^, since we observed a five-fold higher fraction of dead cells after the release (∼25% compared to ∼5% in all other strains (Supplementary Fig. S.9) indicating a severe effect of the deregulation. Mechanistically, the *cln2*Δ*cln3*Δ mutant failed to repress a class of genes linked to the pheromone response even after α-factor was no longer present in the medium. G1 cyclins are known to compete with the pheromone response component Far1, a CDK inhibitor, by targeting it for degradation as their concentrations increase^20,21^. Far1 was initially shown to interact mainly with Cln2^64,65^, later Cln1 was proposed to have a comparable interaction^66^. We here show that Cln2 is more important for silencing the α-factor signaling than Cln1.

In our experiments, the additional perturbations by osmotic stress and α-factor revealed further differences between the mutant strains that would not have been evident from unperturbed cultures. Both perturbations interfere with the cell cycle during G1 phase, which is also the phase most reliant on the action of the analyzed cyclins. Especially, the differences between Cln1 and Cln2 became evident in the stress experiments, for example in the quantitative analysis of the peak times (Fig. 3B). While no effect of the loss of Cln1 alone was visible in the unstressed experiment, the global analysis of all oscillating genes in the stressed mutant showed a delay compared to the wild type, distinct from an even stronger effect in the Cln2 mutant.

Cell cycle synchronization with α-factor is frequently used in cell cycle studies, but is not without effect on the cells after the release (*e.g*.^40,67^ and this study). While we could use this lasting perturbation to identify further differences between functions of the cyclins, some other effects might be masked by the initial decaying response to α-factor. For example, the only slightly altered length of the G1 phases of the Cln1 and Cln2 single and double mutants could be due to the stronger retainment of Whi5 in the nucleus in cells treated with α-factor^67^. The differences in Whi5 localization caused by the lack of Cln1 and Cln2, and therefore their potentially longer G1 phases would not become visible. Accordingly, experimental setups utilizing α-factor synchronization should be handled with care, but can then still be very valuable for analyzing the cell cycle.

## Methods

### Strains

BY4741 (*MAT***a** his3Δ1 leu2Δ0 met15Δ0 ura3Δ0) haploid *S. cerevisiae* strain was used as parental strain. Deletion cassettes for all mutants were generated on a PCR-based protocol using pUG72 (Euroscarf accession number P30117) as template. Deletion cassettes were transformed into BY4741 and deletions were selected on minimal medium lacking uracil. Successful integration was controlled by PCR. The Ura3 selection marker was removed by expression of Cre recombinase from plasmid pSH68 (Euroscarf accession number P30674).

### Growth conditions and synchronization

All experiments were performed in SD medium. For osmostress experiments, 0.4 M NaCl was added. For time course experiments, cells were synchronized as described before^68^ with small modifications. Cells were grown over night and inoculated in fresh medium to OD_600_ = 0,05 and grown until OD_600_ = 0,2. Afterwards, cells were washed, re-suspended in the same volume of fresh media and 5 μg/ml α-factor was added. After 3 hours of synchronization, cells were washed and released in fresh medium, containing 0.4 M NaCl for osmostress experiments.

### Time course, RNA extraction and sample preparation

Samples were taken over 150 min (60 min every 5 min, afterwards every 10 min) and frozen in liquid nitrogen. RNA was extracted with Nucleospin 96 RNA Kit (Machery-Nagel, cat 740466.4) with small modifications. Cell lysis was done for 30 min at 30 °C by adding 450 μl of lysis buffer containing 1 M sorbitol, 100 mM EDTA, and 0.45 ml lyticase (10 IU/ml). The rest of the RNA extraction was performed according to manufacturer’s details. Extracted RNA was converted to cDNA, barcoded and sequenced with Illumina HiSeq 2500.

Additionally, samples were taken over 150 min (for the first 70 min every 10 min, afterwards every 20 min) for flow cytometry, DNA was stained with SYBR green (S9430, Sigma-Aldrich) and DNA content was measured to assess cell cycle progression. Briefly, cells were incubated in 70% Ethanol and washed twice with 50 mM Tris-HCl pH8, RNAse A was added for 40 min and incubated at 37 °C. Again, cells were washed twice with 50 mM Tris-HCl pH 8 and incubated for 1 hour with Proteinase K at 37 °C, followed by two washing steps and resuspended in SYBR green (1:1000) and incubated for 1 hour at room temperature in a dark chamber. Unbound dye was removed by resuspending cells in 50 mM Tris-HCl (pH 8) and cells were sonicated using a Diagenode bioruptor for 3 cycles of 10″ ON and 20″ OFF in low intensity. Afterwards DNA content was analyzed by FACS using BD LSRII system (BD Biosciences).

### RNA sequencing, data processing pipeline

RNA reads were aligned to reference genome using BOWTIE and filtered for rRNAs. Every sequence was normalized for PCR bias using UMIs^69^ and cleaned if the reads align more than once to the genome. Reads were normalized to total expression reads and genes with expression below 10 reads were excluded from the analysis. Time points were removed if total reads were less than 7·10^4^ (Supplementary Fig. S.10). All experiments were done in two independent biological replicates. To test reproducibility Pearson correlation between median expression of all genes was calculated. Since the correlation between replicates was very high (Supplementary Fig. S.11) we merged the experimental data and used the mean of expression.

### Clustering and functional enrichment of clusters

To identify general modes of gene expression over the cell cycle, we performed a clustering analysis on the gene expression time courses. The changes in mRNA expression in the wild type over time were clustered once for unstressed conditions and once for osmostress by k-means clustering. We manually identified all clusters with oscillatory behavior, representing genes, which are regulated during the cell cycle. The genes in these wild type oscillating clusters were plotted for all mutants to assess the changes in the oscillatory behavior between them. To understand which cellular functionalities are associated to the genes in each cluster, the clusters were tested for functional enrichment using GO term enrichment provided by YeastMine database^30^ and via mapping to a generic proteomap^29^, which sorts proteins (here: transcripts) into functional groups and represents them on a clearly structured two-dimensional representation.

### Detection of periodic genes

The MoPS package^25^ for R was used and extended to quantify periodicity for individual genes. The algorithm gives an estimate of how periodic a gene expression time course is, based on a model with several adjustable parameters, which are estimated for each gene: The period length λ, first peak time of the oscillation **φ**, amplitude A, mean expression level m and a decay factor accounting for the loss of synchrony after the release over time. With the optimum parameters for each gene, a periodicity score is calculated, which is, in short, the larger the better the time course complies with the periodic model. In addition to the periodicity score provided by the package, we calculated the Bayesian Information Criterion (BIC) to punish good fits which are based on a time course containing only low numbers of measurement points since the MoPS periodicity score does not explicitly account for the number of data points for each gene. The BIC is a standard method to punish over-fitting. To be more stringent, we neglected genes with less than 13 valid time points, usually the ones with low overall expression. The optional shaping parameter of the MoPS package, which allows the adaptation of the shape of individual parts of the fitted periodic curve, was not used since it caused many false positives. As in Eser *et al*.^25^, we defined a cutoff value for the periodicity score based on the result of the 200 most periodic genes listed in cyclebase^26^ (20% false negative rate). We used the same approach for the BIC normalized by the estimated amplitude of each gene, to select only genes as periodic which have a sufficiently good model fit (Supplementary Fig. S.2). The identified set of oscillating genes shows overlap to previously published studies (Supplementary Fig. S.3).

### Local regression of peak time shifts

The parameters of the MoPS model estimate were calculated for all genes in all mutants and conditions. Between those experiments we could compare the values for the characteristic parameters for all genes and especially for the oscillating subgroup. We focused on the peak time **φ** and analyzed how this time is shifted between wild type and mutants in stressed and unstressed conditions. We therefore plotted the estimated wild type peak times scattered against the peak times of the stressed and unstressed mutants in a two-dimensional plot. The average behavior of peak time shifts in the mutants was visualized using local regression (locally weighted scatterplot smoothing, lowess) to show the trend of the shift of peak times over the entire cell cycle. The Matlab function ‘smooth’ with the option’ rlowess’ was used, performing robust local regression with a first degree polynomial using a span of 20% of the total data points.

### Transcription factor network analyses

We obtained a generic yeast transcription factor (TF) network from the YEASTRACT database^35^, including all TF-target pairs that were identified experimentally by both DNA binding and expression evidence (8685 edges, Supplementary File 3). If the expression of all targets of each transcription factor showed an oscillatory behavior it was again analyzed with MoPS resulting in a periodicity score of the gene expression driven by a specific transcription factor.

### Automatic detection of transcription factor target peak expression

To quantify the length of individual cell cycle phases in the mutants, we identified the peak expression times of selected transcription factor targets. We thereby accounted for the potentially different cell cycle (phase) lengths in the first and second cell cycle, which can be for example due to the lingering effect of α-factor in the first cell cycle. This is not feasible with the MoPS algorithm. To do so expression trajectories of all target genes of a selected transcription factor were averaged and smoothed with a spline interpolant (Matlab interp1 function with option ‘spline’, final resolution of 1 minute). A simple peak detection was implemented by searching for points that are expressed higher than the next and previous 10 points (‘detected peaks’). Thus detected peaks were only considered if they occurred after all peaks of the target genes assigned to the previous cell cycle phase (‘valid peaks’). For cases were several transcription factors define one phase transition, valid peaks’ times were averaged to obtain the phase transition time. To quantify the variability of the α-factor release and to prove reproducibility of our data we applied cell cycle phase classification on the additional data sets obtained from the Barkai group (Supplementary Fig. S.12).

### Quantification of delays via cross correlation

With cross correlation delays between signals can be measured. Thereby, the time point with the highest cross correlation marks the delay; a high absolute value of the correlation indicates similar signals. We used the method to analyze the delays between target expression ‘signals’ of SBF and MBF. Cross correlations were calculated with the MatLab function’crosscorr’ between the pairs of mean target expression (MBF and SBF in one mutant or MBF in a mutant and MBF in the wild type). To obtain mean target expression, each gene trajectory was first normalized to its temporal mean, linearly interpolated to 1 min time steps, then the trajectories of all target genes of MBF or SBF (defined from different sources, Supplementary File 4) were averaged.

### Microscopy and image analysis for bud index calculation

To control synchronization and release from α-factor arrest, a mixed culture with OD_600_ = 0.4 was incubated at 30 C and synchronized with 5 μg/ml α-factor in SD medium for 3 h (time point “syn.” in Fig. 6C). Afterwards α-factor was removed by washing samples twice with SD. Samples were taken every 10 min for 150 min, fixed for 45 min in 4% paraformaldehyde and washed twice with PBS. Microscopic images were taken using a Olympus IX83 inverted microscope in z-stacks. Brightfield images were acquired with an iXon EMCCD Cameras, ANDOR. Maximum intensity projections were calculated. Detection of bud emergence was performed manually utilizing BudJ (http://www.ibmb.csic.es/groups/spatial-control-of-cell-cycle-entry). For each time point more than 200 cells were counted and classified into three groups: Cells, buds and large daughter cells which from the brightfield images could not be clearly identified as separated from their mothers, but are to large to be newly formed buds. Those cells were distributed equally to the cell and bud group for the calculation of the bud index. The resulting uncertainty is shown as shaded area in Fig. 6C (upper bound: all “large buds” counted as buds, lower bound: all “large buds”counted as cells).

## Supplementary information

Supplementary Material

Dataset 1

Dataset 2

Dataset 3

Dataset 4

## Data Availability

GO enrichment^30^ for k-means clusters is available in Supplementary File 1. All oscillating genes obtained by MoPS algorithm^25^, including the obtained parameters are available in Supplementary File 2. Transcription factor and targets from YEASTRACT^35^ are listed in Supplementary File 3. Supplementary File 4 concludes target genes for MBF and SBF for the different data sets^35,39,40,49^. Raw sequencing data are available at https://www.ncbi.nlm.nih.gov/sra/SRP151525.
