# Supplementary material for: A transcriptome-wide analysis deciphers distinct roles of G1 cyclins in temporal organization of the yeast cell cycle
Source: Sci Rep. 2019 Mar 4;9:3343. doi: 10.1038/s41598-019-39850-7 (PMC6399449; doi:10.1038/s41598-019-39850-7)
Supplement: Supplementary file 1 — Supplementary Material [file 41598_2019_39850_MOESM1_ESM.pdf]

A transcriptome-wide analysis deciphers distinct roles of G1 cyclins in temporal organization of the yeast cell cycle

Supplementary Material

Lotte Teufel[1,2°], Katja Tummler[1°], Max Flöttmann[1] , Andreas Herrmann[2] , Naama Barkai[3], Edda Klipp[1*]

[1] Theoretical Biophysics, Humboldt-Universität zu Berlin
[2] Molecular Biophysics, Humboldt-Universität zu Berlin
[3] Weizmann Institute of Science, Rehovot

° Equal contribution
corresponding author: Edda Klipp, edda.klipp@rz.hu-berlin.de

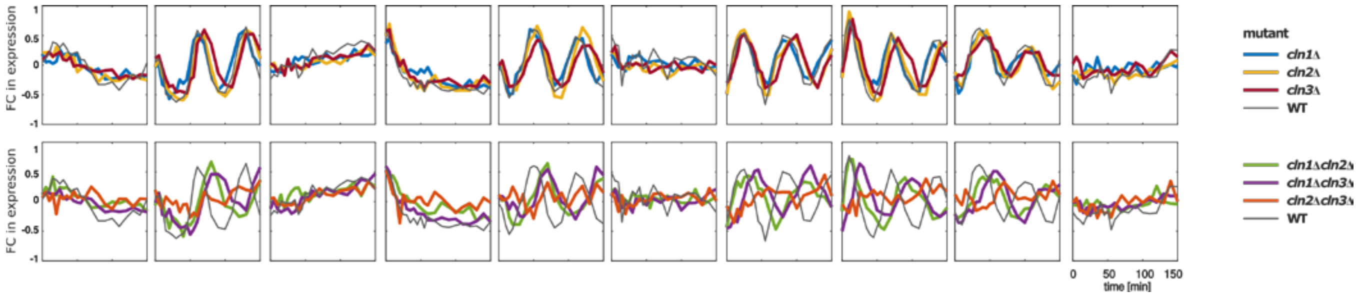

Figure S.1: Overview of differentially expressed gene clusters found by k-mean clustering on wild type (WT) gene expression trajectories under unstressed growth conditions. Differentially expressed genes in wild type are plotted for all strains to assess differences in gene expression. Upper panel represent single deletions and lower panel represents double deletion mutants. Mean expression is shown for wild type in gray and corresponding genes in the mutants strains are plotted in colors.

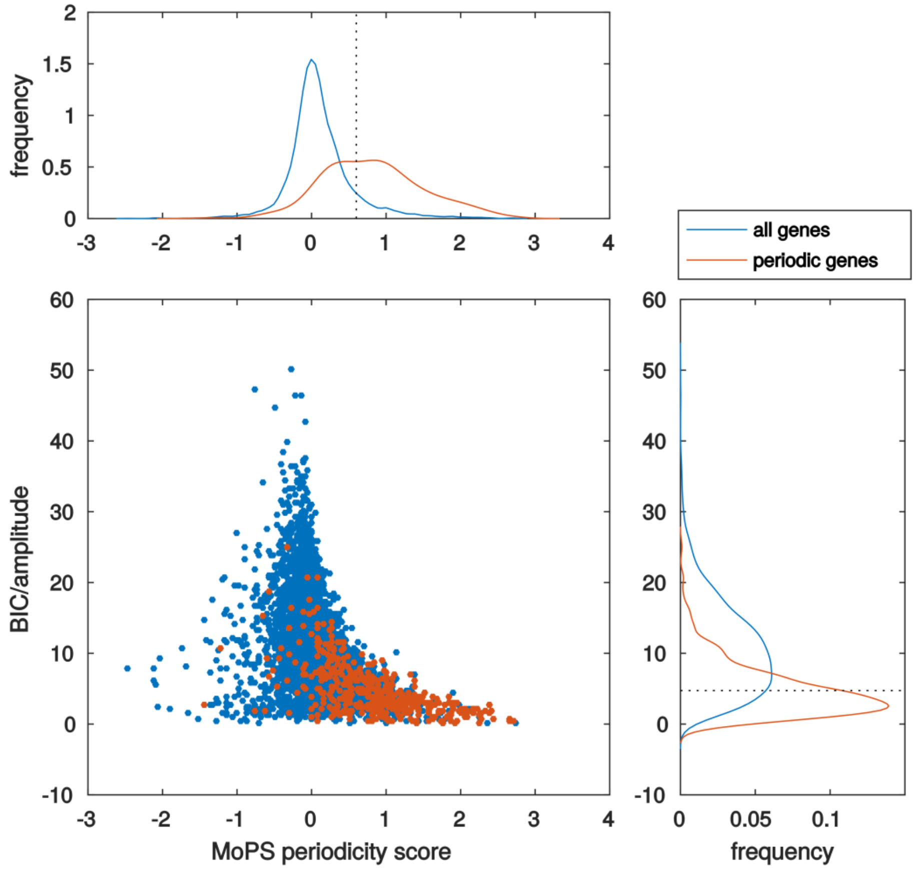

Figure S.2: Calculated MoPS periodicity score and BIC. Blue dots are all yeast genes, orange ones the top 200 oscillating genes from cyclebase [1]. Dotted lines denote the 20% quantiles of the period genes' distributions and was used as cut-off value.

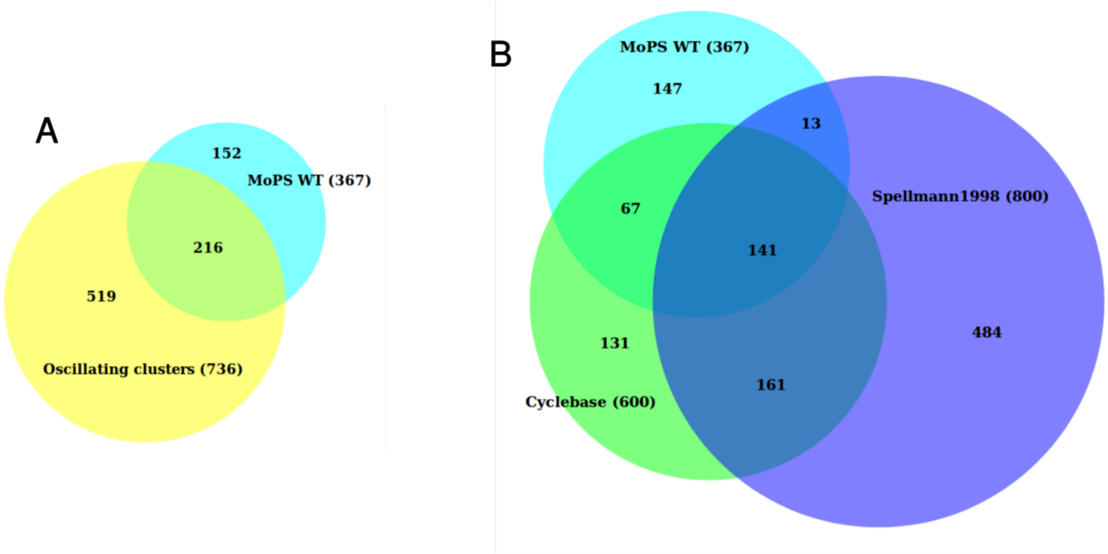

Figure S.3: Comparison of the found sets of oscillating genes. (A) Genes in the 5 oscillating clusters (Figure 2) and oscillating genes identified by MoPS [2]. **(B)** MoPS list compared to previously defined oscillating gene sets (the 500 most oscillating genes from cyclebase [1] and from Spellman *et al.* [3]. Visualization by BioVenn [4].

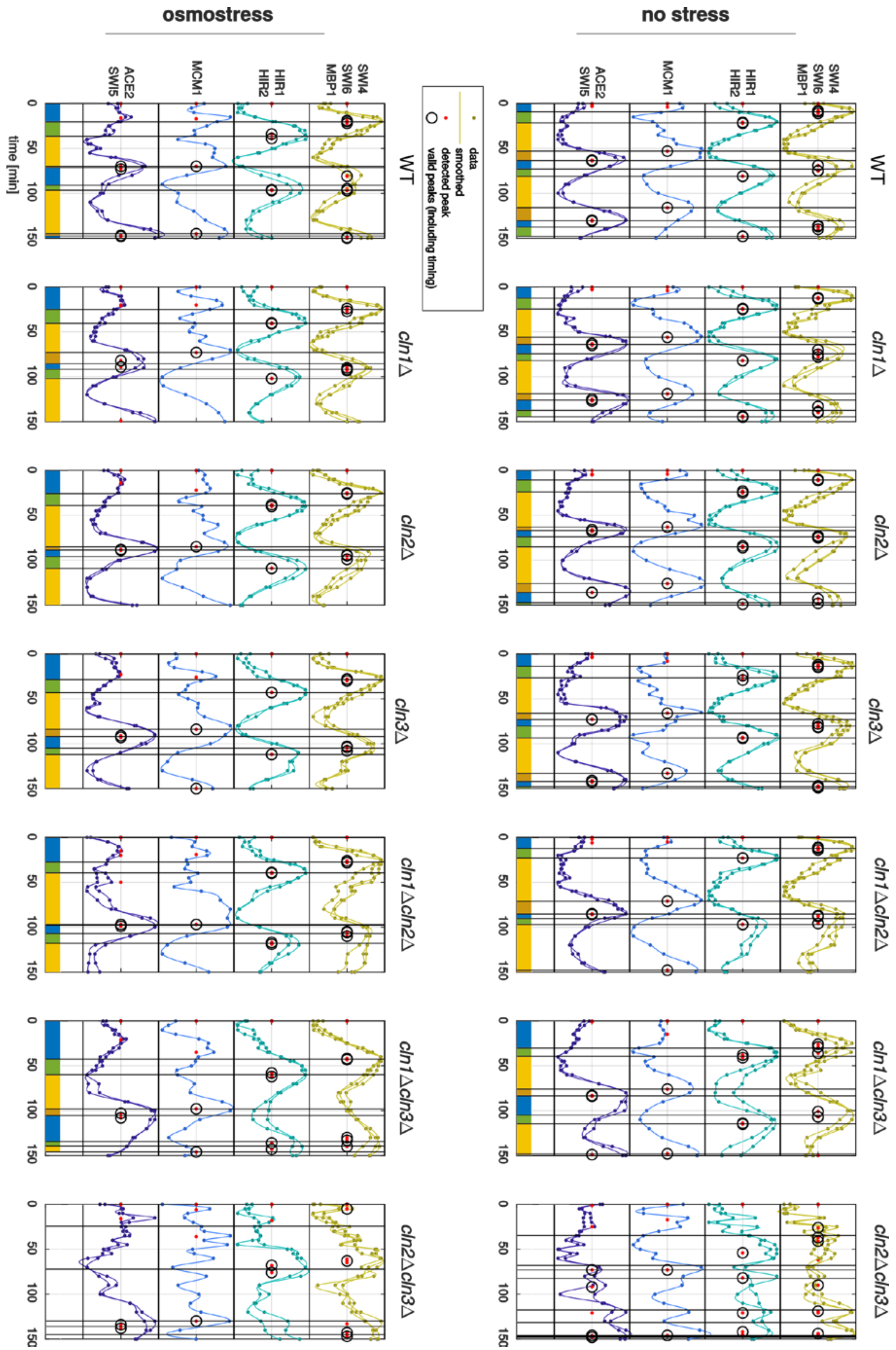

Figure S.4: Expression patterns for target genes for selected "classification set" transcription factors used to define cell cycle phase under all experimental conditions (as shown in detail for the wild type in Figure 4). Trajectories depict mean expression level of the targets (represented as log fold change relative to mean expression of all genes). Phase transitions were defined according to the rules in Supplementary Table S.2 (blue = G1, green = S, light yellow = G2, dark yellow = M, grey = end not detectable).

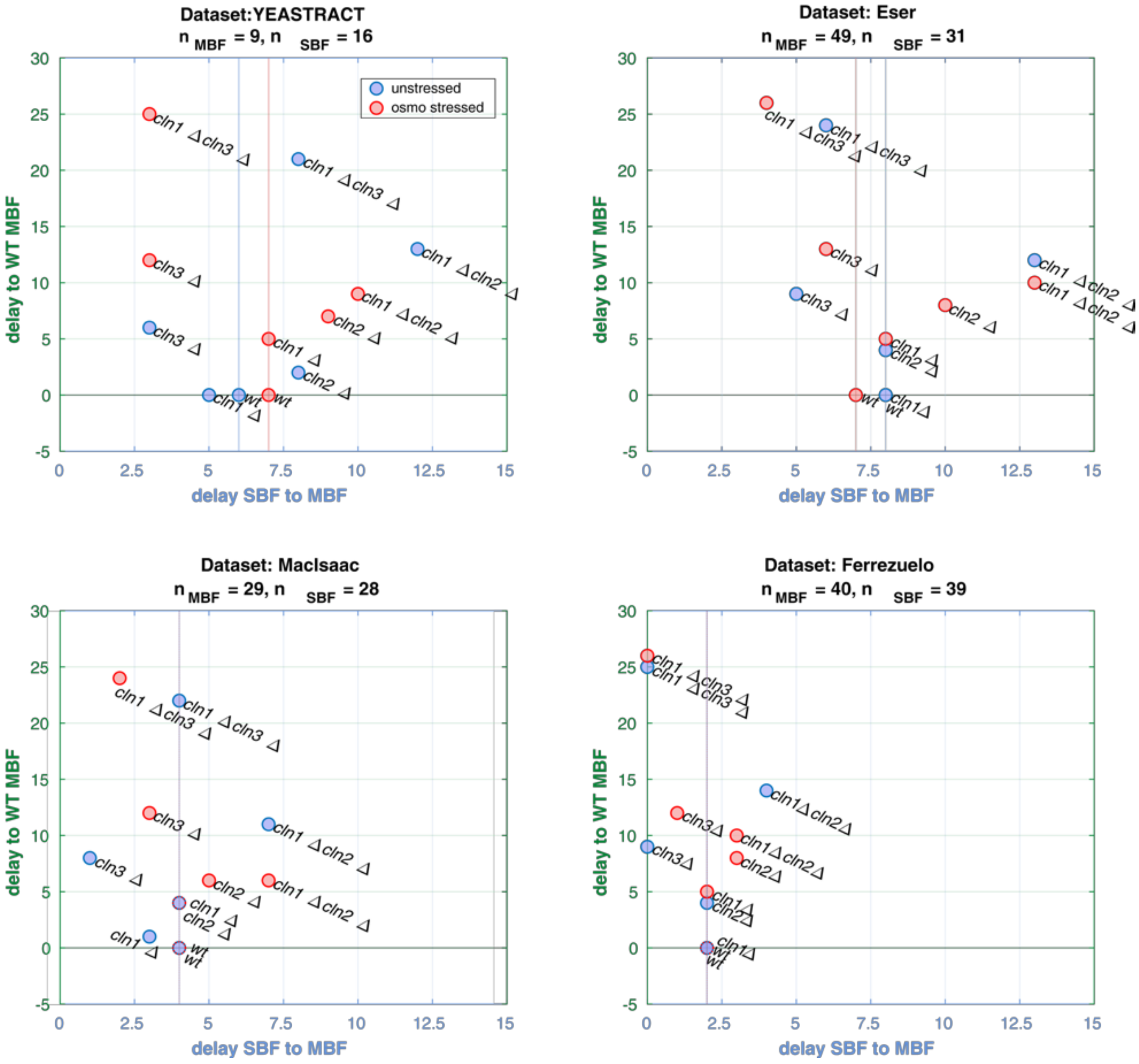

Figure S.5: Delays in expression based on different target gene lists for SBF and MBF for all mutants under both unstressed (blue dots) and osmotic stress (red dots) conditions. The delay between SBF and MBF is shown on the x-axis and the delay of the mutants to the wild type expression is depicted on the y-axis. Analysis is the same as in the main Text Figure 5, with target gene lists as defined by YEASTRACT[5], Eser *et al.* [6], MacIsaac *et al.* [7] and Ferrezuelo *et al.* [8].

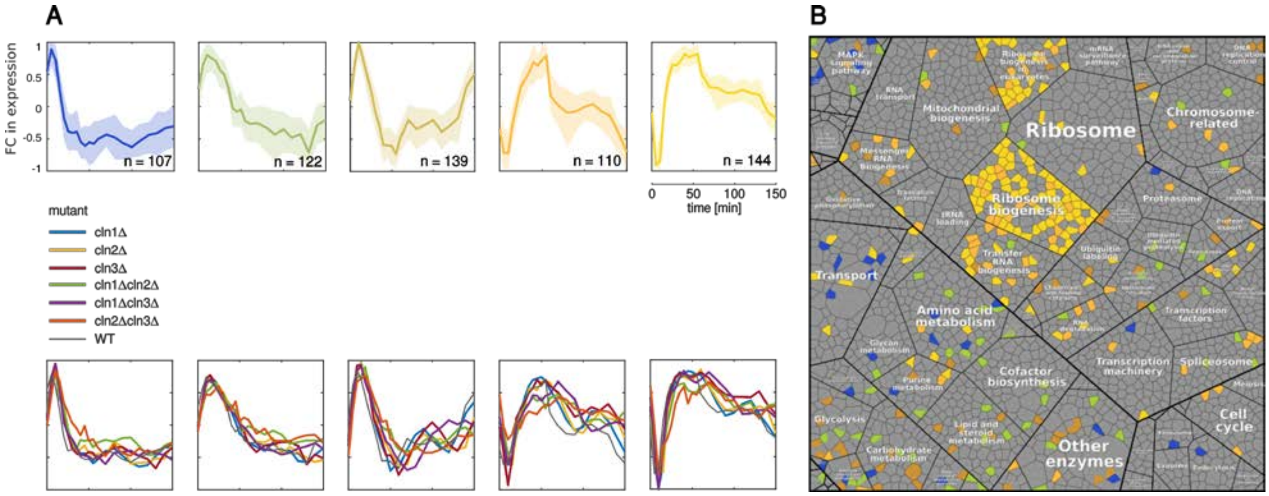

Figure S.6: Acute osmotic stress response. (A) Wild type expression of stress responsive gene clusters (upper panel, line represents mean, shaded area 25% and 75% quantiles). The lower panel shows the mean expression of the corresponding gene clusters in the mutant strains. The wild type behavior is plotted for comparison. **(B)** Functional classification is based on a proteomap [9], genes are colored according to the stress cluster in A.

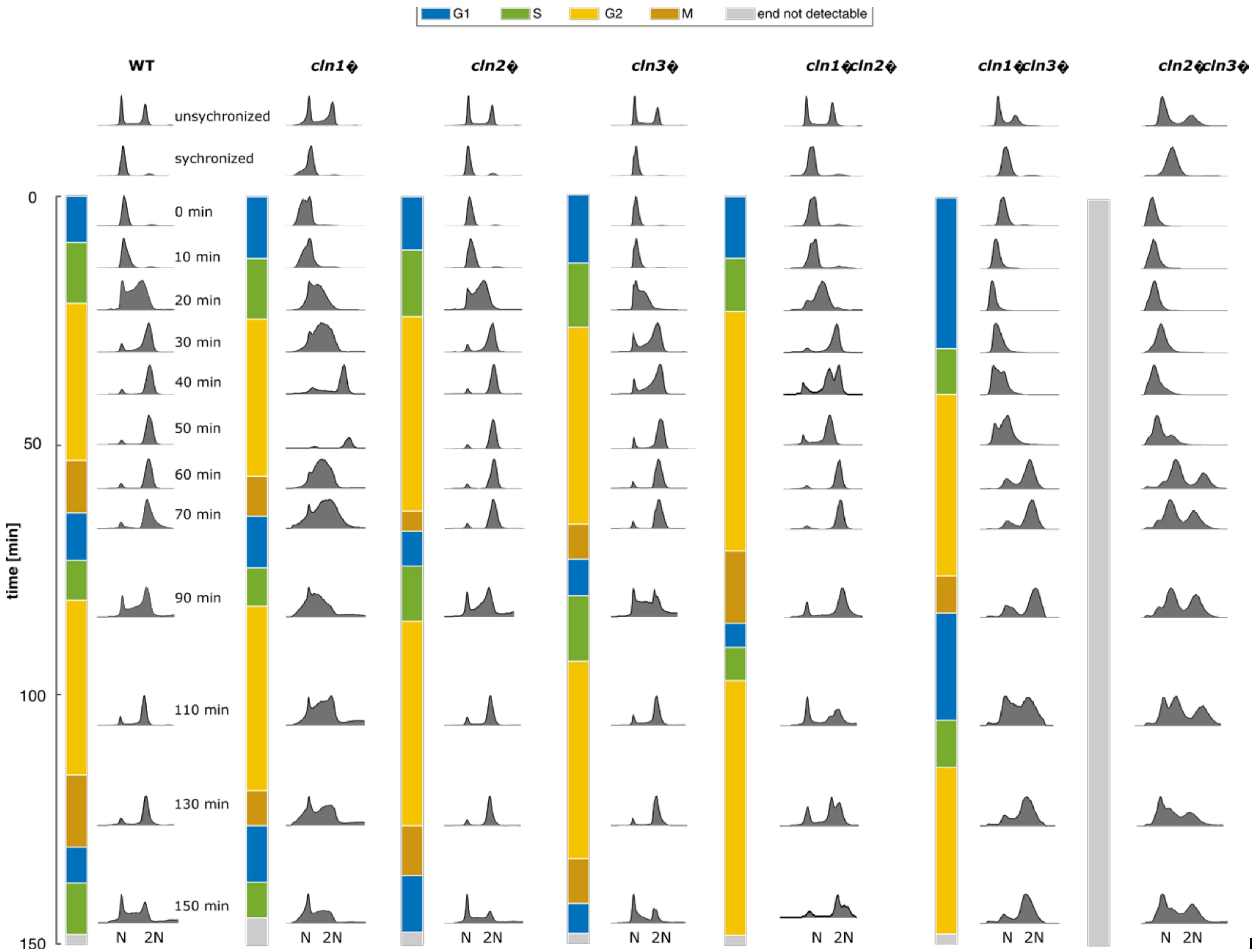

Figure **S.7: Cell cycle progression after α-factor release for all strains.** Colored bars represent cell cycle classification obtained by transcription factor target expression (blue = G1, green = S, light yellow = G2, dark yellow = M, grey = end not detectable). DNA content profile measured by flow cytometry are shown on the right side to each bar.

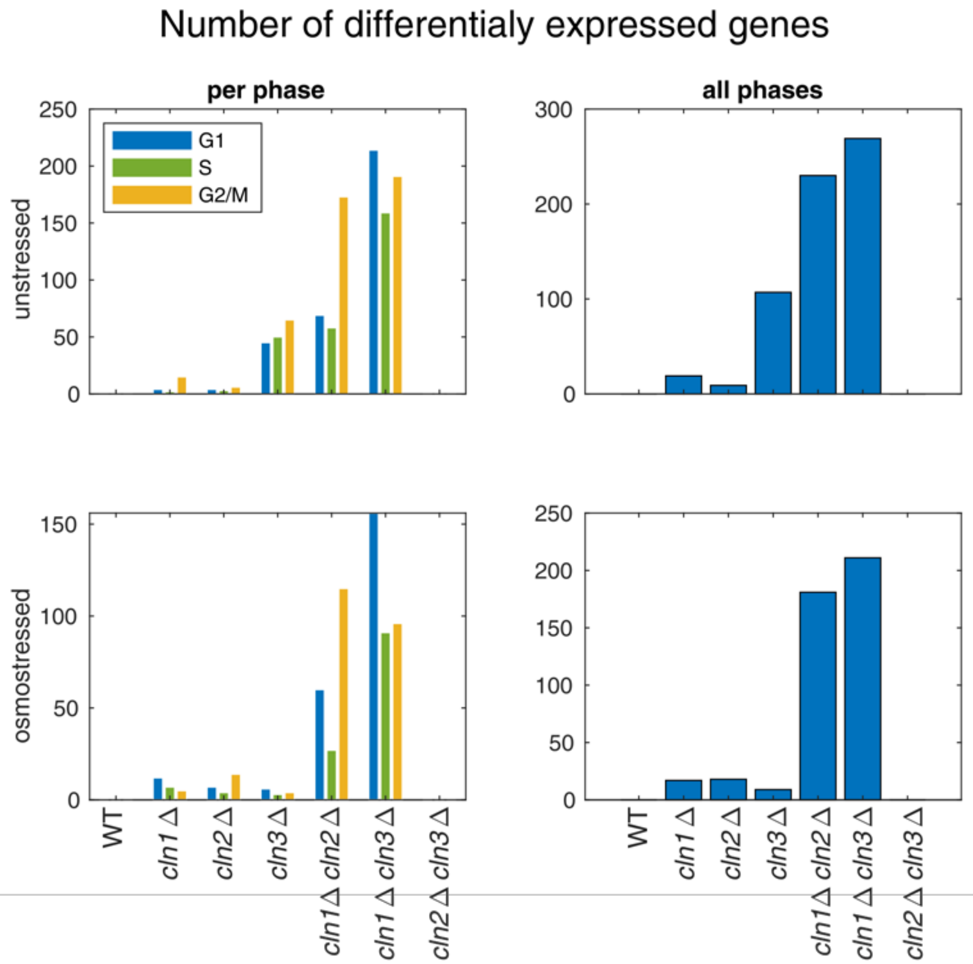

Figure S. 8: Number of differentially expressed genes in the mutants per cell cycle phase. Expression trajectories of the mutants were normalized by scaling corresponding phases to the wild type (WT) phase durations. Samples taken at switching times between the phases were assigned to the previous phase. To calculate differential expression, WT expression was linearly interpolated at the now normalized mutant time points.

Genes were deemed differentially expressed if at least n_ρ = 3 time points were changed more than 3.36-fold ($\log_2(1.75)$) or a low p-value in a Kolmogorov-Smirnow-test compared to the WT expression ($\rho<10^{-8}$ for n_ρ = 0, $\rho<8\times10^{-5}$ for n_ρ =1 and $\rho<0.002$ for n_ρ = 2).

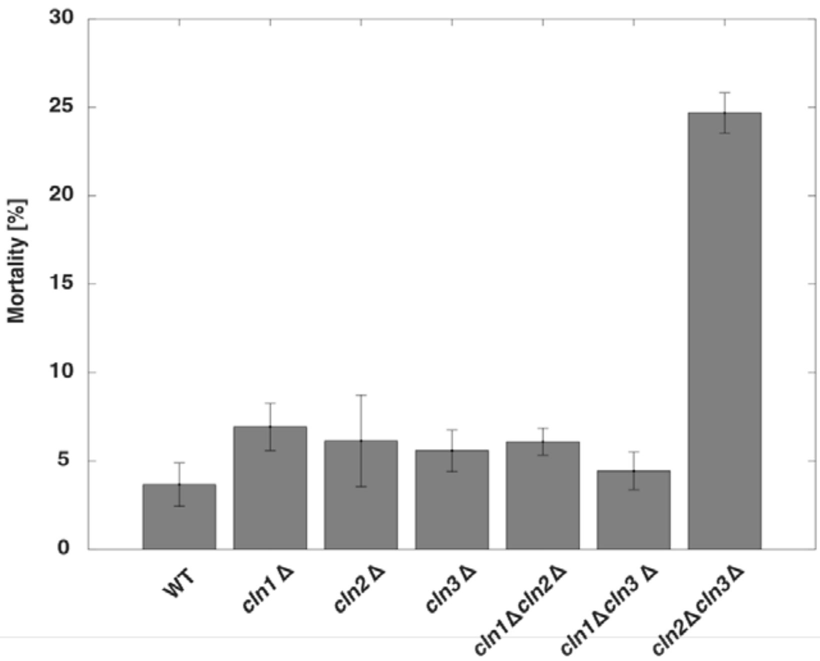

Figure S. 9: Viability test, cells were stained with propidium iodide and percentage of dead cells was measured using Flow cytometry. Propidium iodide can only enter in dead cells and fluorescence can be meascured by FACS. Cells were synchronized (as described in Material&Methods) and samples were taken every 15 min over 150 min. Since no differences in viability over time were detected, mean viability in percent and standard deviation for each strain are depicted.

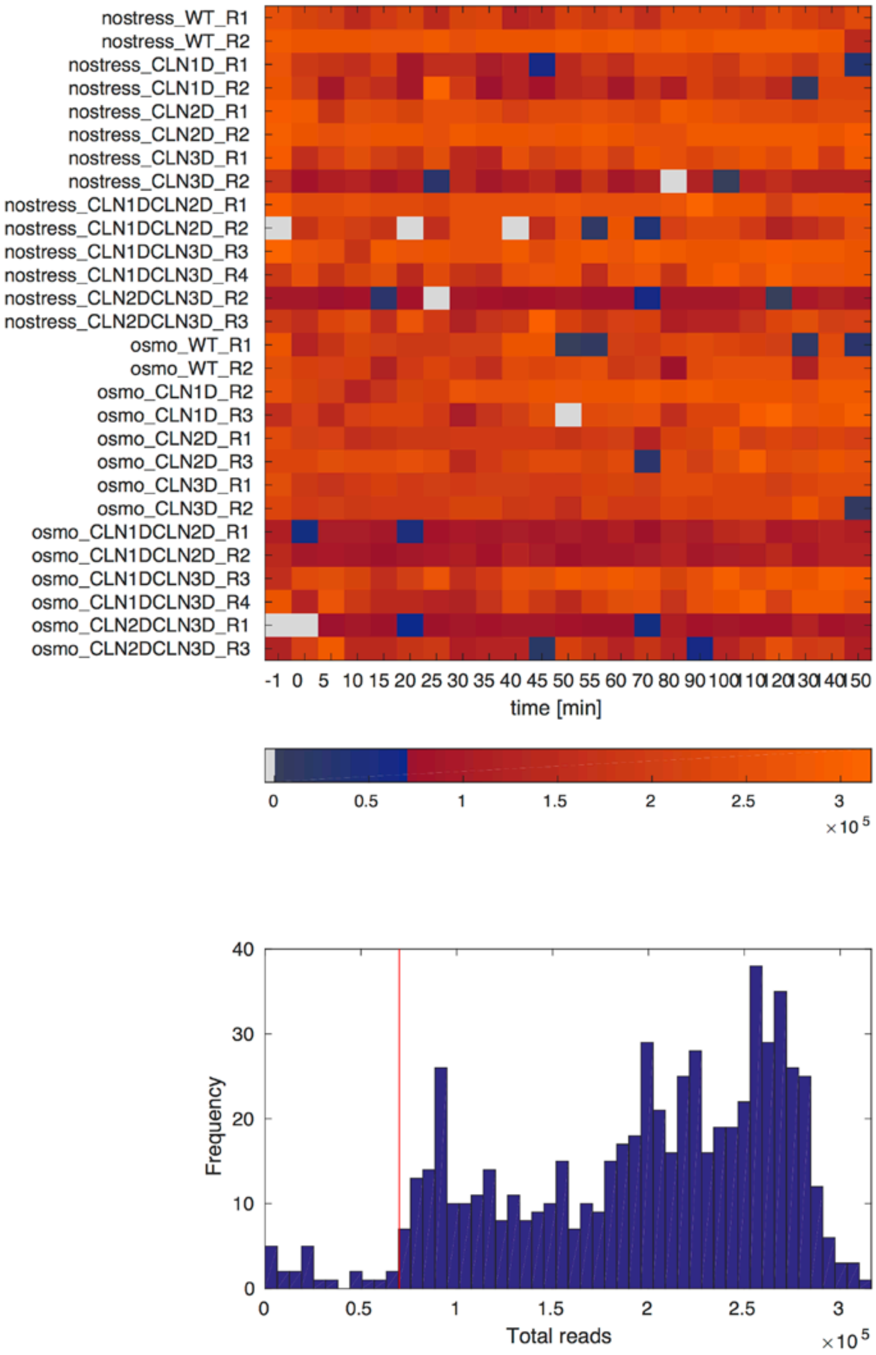

Figure S. 10: Statistics of total reads per sample. Shown are total reads per sample in individual RNAseq experiments over time. Samples with less than 7 x10^4 reads were excluded from the analysis (blue fields in the heatmap plot, red line in histogram). Note that in the histogram missing samples are counted in the first bin, whereas they are specifically shown in gray in the heatmap plot.

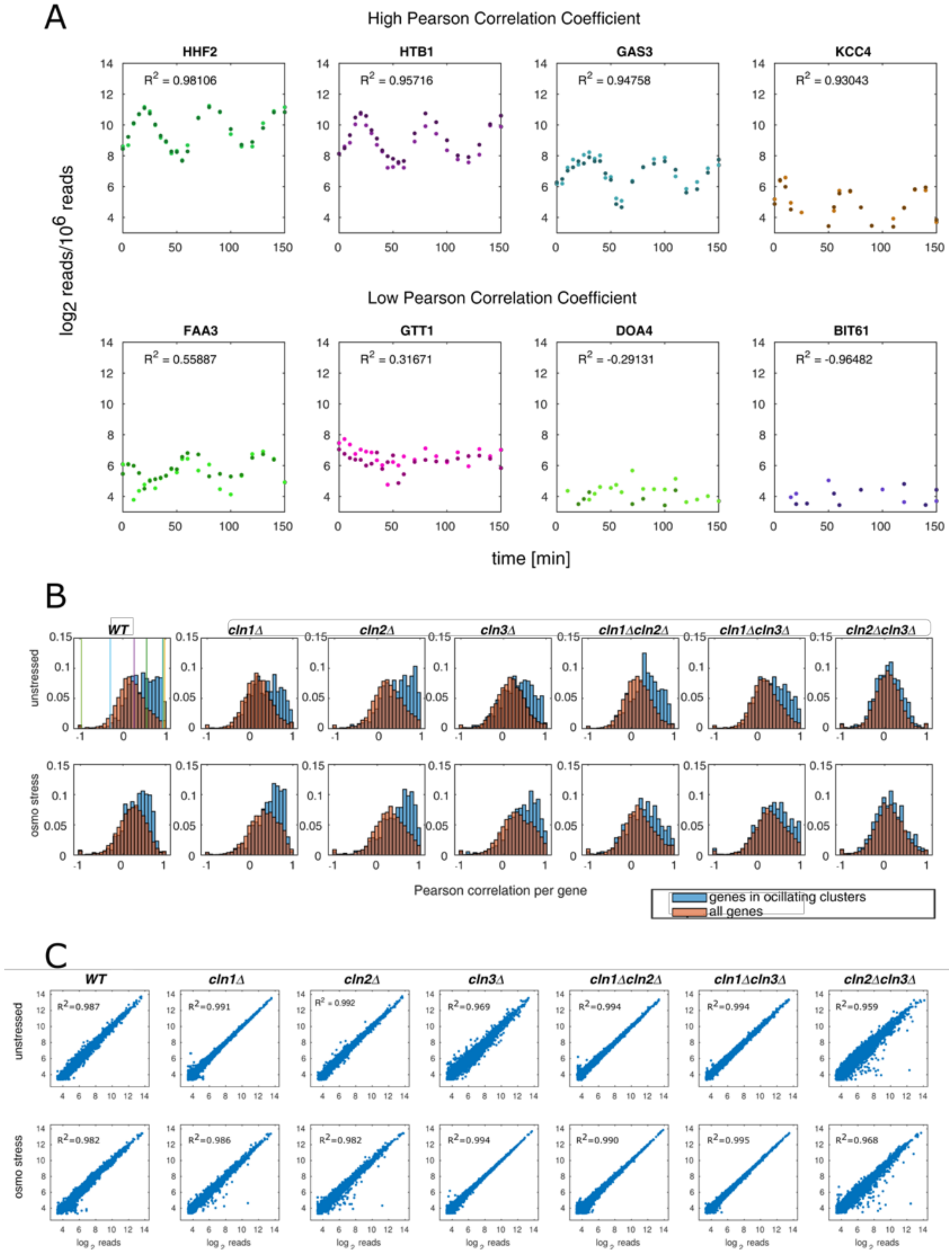

Figure S. 11: Reproducibility of the RNAseq replicates used for the analyses. **(A)** Replicates are similar for individual genes. Data of both replicates for selected genes in the unstressed wild type. Each replicate is shown as light or darker colored dots, along with Pearson correlation coefficient between the replicates. For the genes in the lower panel the correlation coefficient is low, due to the higher noise for genes with lower expression and less regulation during the cell cycle. Nevertheless, the temporal behavior and mean expression in the replicates is similar, which is quantified for the entire data set below. **(B)** Oscillating genes show high correlation between the replicates. Histograms of correlation coefficients between the two replicates for all genes measured in each mutant. The genes from A marked in the respective colors in the unstressed wild type distribution. Genes that are regulated during the cell cycle (here that belong to the oscillating clusters described in the main text, blue histogram) show a higher overall correlation than the entire transcriptome (red histogram). **(C)** Expression means are well preserved between the replicates. The scatterplot of the temporal means of all genes between the two replicates (replicate 1 on the x-axis, replicate 2 on the y-axis) shows a high correlation (>0.95) for all experiments.

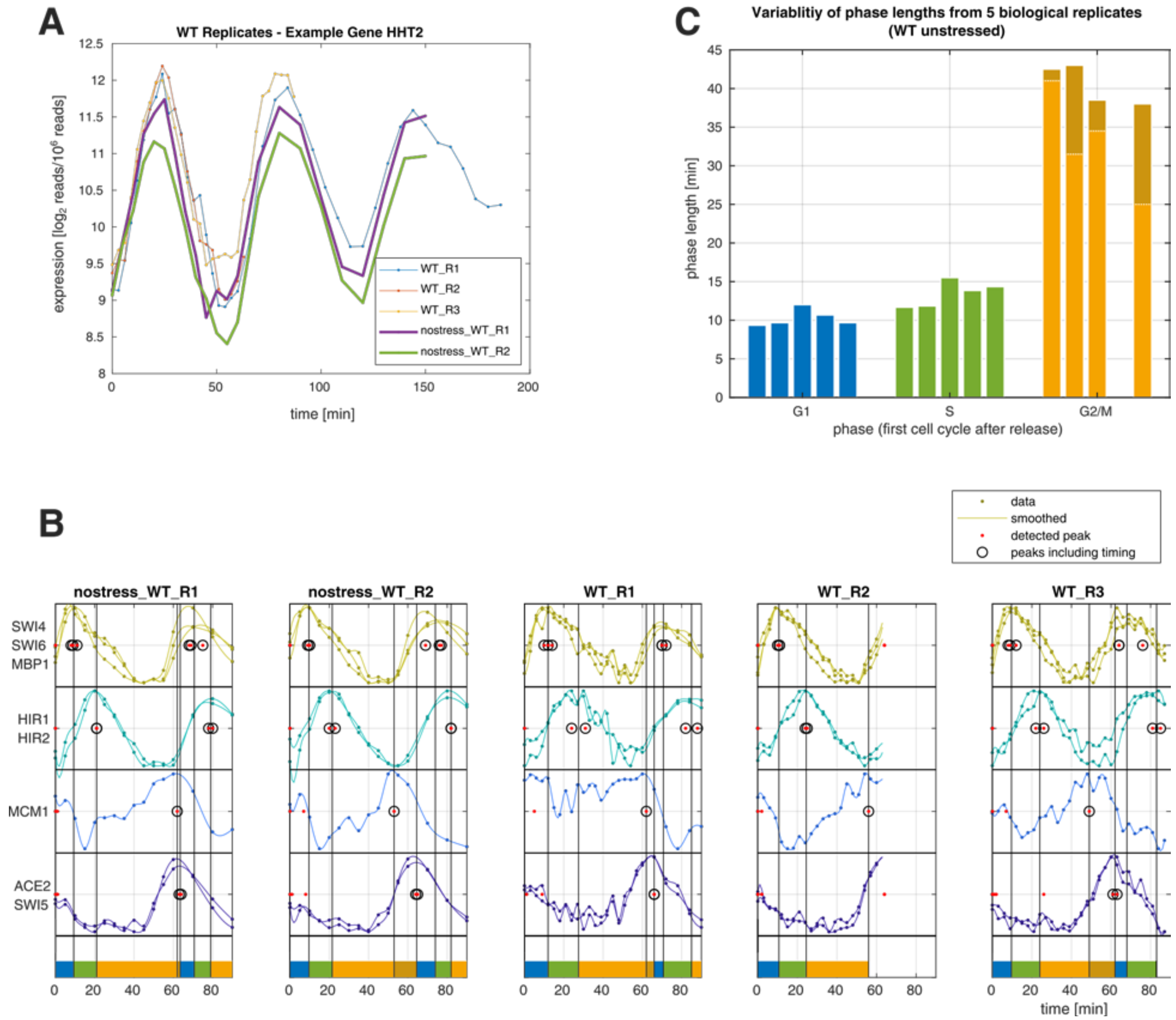

Figure S. 12: Additional replicates of the unstressed wild type quantify variability in the cell cycle phase length calculation and reproducibility of the release. In addition to the two biological replicates used throughout the study (nostress_WT_Rx), three additional biological replicates are shown (WT_Rx) originating from a different set of experiments. (**A**) Expression of an example gene for all 5 biological replicates. (**B**) Cell cycle phase classification as in main text figure 4 for the replicates. Please note that the analysis was only carried out for the time span for which data for all replicates was available (up to 63 minutes). (**C**) Durations of cell cycle phase of the first cell cycle after α-factor release for the 5 biological WT replicates (phases are shown in colors, G2 and M phase are lumped together blue = G1, green = S, light yellow = G2, dark yellow = M).

Table 1: Transcription factors with oscillating target expression. The lower factors are not shown in Figure 4 if they have less than 3 target genes (f) or are not connected to any other transcription factor (u).

ID	ORF	MoPS score	MoPS φ	# targets	short description (SGD)	why not in network
DIG1	YPL049C	0.81	0	6	MAP kinase-responsive inhibitor of the Ste12p transcription factor	
PHD1	YKL043W	1.77	3	32	Transcriptional activator that enhances pseudohyphal growth	
SOK2	YMR016C	1.06	3	375	Nuclear protein that negatively regulates pseudohyphal differentiation	
MSN2	YMR037C	0.93	5	693	Stress-responsive transcriptional activator	
PHO4	YFR034C	1.27	5	65	Basic helix-loop-helix (bHLH) transcription factor of the myc-family	
TEC1	YBR083W	1.34	5	309	Transcription factor targeting filamentation genes and Ty1 expression	
STE12	YHR084W	1.26	6	937	Transcription factor that is activated by a MAPK signaling cascade	
MSS11	YMR164C	1.07	7	9	Transcription factor	
TUP1	YCR084C	0.81	7	109	General repressor of transcription	
CIN5	YOR028C	0.92	8	201	Basic leucine zipper (bZIP) transcription factor of the yAP-1 family	
MBP1	YDL056W	1.66	8	60	Transcription factor	
ASH1	YKL185W	1.04	9	64	Component of the Rpd3L histone deacetylase complex	
MGA2	YIR033W	1.04	12	13	ER membrane protein involved in regulation of OLE1 transcription	
SWI4	YER111C	2.14	12	134	DNA binding component of the SBF complex (Swi4p-Swi6p)	
HAC1	YFL031W	1.2	13	9	Basic leucine zipper (bZIP) transcription factor (ATF/CREB1 homolog)	
SWI6	YLR182W	2.09	13	41	Transcription cofactor	
HIR2	YOR038C	1.71	22	8	Subunit of HIR nucleosome assembly complex	
HCM1	YCR065W	1.16	24	10	Forkhead transcription factor	
FKH2	YNL068C	1.12	33	23	Forkhead family transcription factor	
HMS1	YOR032C	0.79	44	7	bHLH protein with similarity to myc-family transcription factors	
MIG2	YGL209W	1.04	46	4	Zinc finger transcriptional repressor	
MCM1	YMR043W	2.17	55	115	Transcription factor	
YOX1	YML027W	1.31	59	97	Homeobox transcriptional repressor	
SWI5	YDR146C	1.13	61	83	Transcription factor that recruits Mediator and Swi/Snf complexes	
ACE2	YLR131C	1.08	62	116	Transcription factor required for septum destruction after cytokinesis	
YHP1	YDR451C	1.21	62	38	Homeobox transcriptional repressor	
FLO8	YER109C	0.94	63	37	Transcription factor	
CUP9	YPL177C	0.78	65	16	Homeodomain-containing transcriptional repressor	
HIR1	YBL008W	1.5	20	1	Subunit of the HIR complex	f
YRR1	YOR162C	0.88	22	2	Zn2-Cys6 zinc-finger transcription factor	f
AZF1	YOR113W	1	52	2	Zinc-finger transcription factor	f
RLM1	YPL089C	1.28	3	34	MADS-box transcription factor	u
MAL33	YBR297W	1.33	10	11	MAL-activator protein	u
UGA3	YDL170W	2.11	13	4	Transcriptional activator for GABA-dependent induction of GABA genes	u
PIP2	YOR363C	1.01	63	44	Autoregulatory, oleate-activated transcription factor	u
HAL9	YOL089C	0.88	69	8	Putative transcription factor containing a zinc finger	u

Table 2: Overview of selected transcription factors for definition of cell cycle phase duration.

Group	Transcription factor	Periodicity score WT	Estimated target peak time WT [min]	Event at peak (from literature)	References
I	Swi4	2.14	12	G1/S transition	McIntosh *et al*, 2000; Iyer *et al*, 2001; Simon *et al*, 2001; Lee *et al*, 2002; Teixeira *et al*, 2006; Ferrezuelo *et al*, 2009
	Swi6	2.09	13		
	Mbp1	1.66	8		
II	Hir1	1.5	20	S/G2 transition	Sherwood *et al*, 1993; Spector *et al*, 1997
	Hir2	1.71	22		
III	Mcm1	2.17	55	G2/M transition	Loy *et al*, 1999; Kumar *et al*, 2000
IV	Swi5	1.13	61	Mitotic exit, reset for new G1	Dohrmann *et al*, 1992, 1996; McBride *et al*, 1999; Laabs *et al*, 2003
	Ace2	1.08	61		

Table 3: Group of α-factor induced genes with higher expression over the experiment in *cln2Δcln3Δ* (also see Figure 6)

ORF	ID	Name	brief description (SGD)	mean expression WT	mean expression *cln2Δcln3Δ*	fold change
YNR044W	AGA1	a-AGglutinin	Anchorage subunit of a-agglutinin of a-cells	7.56	8.89	2.50
YHR030C	SLT2	Suppressor of the LyTic phenotype	Serine/threonine MAP kinase	7.15	8.57	2.68
YGR032W	GSC2	Glucan Synthase of Cerevisiae	Catalytic subunit of 1.3-beta-glucan synthase	6.51	7.94	2.70
YIL015W	BAR1	BARrier to the alpha factor response	Aspartyl protease	6.22	7.81	3.02
YPL192C	PRM3	Pheromone-Regulated Membrane protein	Protein required for nuclear envelope fusion during karyogamy	6.07	7.67	3.04
YCR089W	FIG2	Factor-Induced Gene	Cell wall adhesin. expressed specifically during mating	7.70	9.41	3.26
YDR085C	AFR1	Alpha-Factor Receptor regulator	Protein required for pheromone-induced projection (shmoo) formation	5.60	7.34	3.34
YIL117C	PRM5	Pheromone-Regulated Membrane protein	Pheromone-regulated protein. predicted to have 1 transmembrane segment	5.98	7.84	3.64
YNL279W	PRM1	Pheromone-Regulated Membrane protein	Pheromone-regulated multispanning membrane protein	6.81	8.72	3.77
YGL032C	AGA2	a-AGglutinin	Adhesion subunit of a-agglutinin of a-cells	7.09	9.02	3.81
YBR040W	FIG1	Factor-Induced Gene	Integral membrane protein required for efficient mating	6.40	8.40	3.99
YMR232W	FUS2	cell FUSion	Cell fusion regulator	5.08	7.12	4.12
YJL170C	ASG7	a-Specific Gene	Protein that regulates signaling from G protein beta subunit Ste4p	7.07	9.18	4.32
YLR452C	SST2	SuperSensiTive	GTPase-activating protein for Gpa1p	5.57	7.76	4.56
YDR461W	MFA1	Mating Factor A	Mating pheromone a-factor	8.67	11.06	5.21
YMR096W	SNZ1	SNooZe	Protein involved in vitamin B6 biosynthesis	5.76	8.71	7.72

Reference:

1. Santos, A., Wernersson, R. & Jensen, L. J. Cyclebase 3.0: A multi-organism database on cell-cycle regulation and phenotypes. *Nucleic Acids Res.* **43,** D1140–D1144 (2014).
2. Eser, P. *et al.* Periodic mRNA synthesis and degradation co-operate during cell cycle gene expression. *Mol. Syst. Biol.* **10,** (2014).
3. Spellman, P. *et al.* Comprehensive Identification of Cell Cycle regulated Genes of the Yeast Saccharomyces cerevisiae by Microarray Hybridization. *Mol. Biol. Cell* **9,** 3273–3297 (1998).
4. Hulsen, T., de Vlieg, J. & Alkema, W. BioVenn - A web application for the comparison and visualization of biological lists using area-proportional Venn diagrams. *BMC Genomics* **9,** (2008).
5. Teixeira, M. C. *et al.* The YEASTRACT database: An upgraded information system for the analysis of gene and genomic transcription regulation in Saccharomyces cerevisiae. *Nucleic Acids Res.* **42,** (2014).
6. Eser, U., Falleur-Fettig, M., Johnson, A. & Skotheim, J. M. Commitment to a Cellular Transition Precedes Genome-wide Transcriptional Change. *Mol. Cell* **43,** 515–527 (2011).
7. Macisaac, K. D. *et al.* An improved map of conserved regulatory sites for Saccharomyces cerevisiae. *BMC Bioinformatics* **7,** (2006).
8. Ferrezuelo, F., Aldea, M. & Futcher, B. Bck2 is a phase-independent activator of cell cycle-regulated genes in yeast. *Cell Cycle* **8,** 239–252 (2009).
9. Liebermeister, W. *et al.* Visual account of protein investment in cellular functions. *Proc. Natl. Acad. Sci. U. S. A.* **111,** 8488–93 (2014).
10. McIntosh, P. B., Taylor, I. A., Frenkiel, T. A., Smerdon, S. J. & Lane, A. N. The influence of DNA binding on the backbone dynamics of the yeast cell- cycle protein Mbp1. *J. Biomol. NMR* **16,** 183–196. (2000).
11. Iyer, V. R. *et al.* Genomic binding sites of the yeast cell-cycle transcription factors SBF and MBF. *Nature* **409,** 533–538 (2001).
12. Simon, I. *et al.* Serial regulation of transcriptional regulators in the yeast cell cycle. *Cell* **106,** 697–708 (2001).
13. Lee, T. I. *et al.* Transcriptional regulatory networks in Saccharomyces cerevisiae. *Science (80-.).* **298,** 799–804 (2002).
14. Teixeira, M. C. *et al.* The YEASTRACT database: a tool for the analysis of transcription regulatory associations in Saccharomyces cerevisiae. *Nucleic Acids Res.* **34,** 446–451 (2006).
15. Sherwood, P. W., V-m Tsang, S. & ANN OSLEYl, M. Characterization of HIR1and HIR2, Two Genes Required for Regulation of Histone Gene Transcription in Saccharomyces cerevisiae. *Mol. Cell. Biol.* **13,** 28–38 (1993).
16. Spector, M. S. *et al.* Hir1p and Hir2p function as transcriptional corepressors to regulate histone gene transcription in the Saccharomyces cerevisiae Hir1p and Hir2p Function as Transcriptional Corepressors To Regulate Histone Gene Transcription in the Saccharomyces cerevisiae. *Microbiology* **17,** 545–552 (1997).
17. Loy, C. J., Lydall, D. & Surana, U. NDD1, a high-dosage suppressor of cdc28-1N, is essential for expression of a subset of late-S-phase-specific genes in Saccharomyces cerevisiae. *Mol. Cell. Biol.* **19,** 3312–3327 (1999).
18. Kumar, R. *et al.* Forkhead transcription factors, Fkh1p and Fkh2p, collaborate with Mcm1p to control transcription required for M-phase. *Curr. Biol.* **10,** 896–906 (2000).
19. Dohrmann, P. R. *et al.* Parallel pathways of gene regulation: homologous regulators SWI5 and ACE2 differentially control transcription of HO and chitinase. *Genes Dev.* **6,** 93–104 (1992).
20. Dohrmann, P. R., Voth, W. P. & Stillman, D. J. Role of negative regulation in promoter specificity of the homologous transcriptional activators Ace2p and Swi5p. *Mol. Cell. Biol.* **16,** 1746–58 (1996).
21. McBride, H. J., Yu, Y. & Stillman, D. J. Distinct regions of the Swi5 and Ace2 transcription factors are required for specific gene activation. *J Biol Chem* **274,** 21029–36 (1999).
22. Laabs, T. L. *et al.* ACE2 is required for daughter cell-specific G1 delay in Saccharomyces cerevisiae. *Proc. Natl. Acad. Sci. U. S. A.* **100,** 10275–10280 (2003).
